# Estradiol Priming Potentiates the Kisspeptin-Induced Release of LH in Ovariectomized Cows

**DOI:** 10.3390/ani11051236

**Published:** 2021-04-25

**Authors:** Gustavo Guerino Macedo, Emiliana de Oliveira Santana Batista, Gustavo Martins Gomes dos Santos, Michael J. D’Occhio, Pietro Sampaio Baruselli

**Affiliations:** 1 Faculty of Veterinary Medicine and Animal Sciences, Federal University of Mato Grosso do Sul, Campo Grande 79070-900, Mato Grosso do Sul, Brazil; 2 Faculty of Veterinary Medicine and Animal Science, University of Sao Paulo, Sao Paulo 05508-270, Sao Paulo, Brazil; emilianamili@hotmail.com; 3 Sheepembryo, Londrina 80010-000, Parana, Brazil; gugamgs@hotmail.com; 4 Faculty of Science, School of Life and Environmental Sciences, The University of Sydney, Sydney, NSW 2006, Australia; michael.docchio@sydney.edu.au

**Keywords:** estradiol priming, kisspeptin, LH, fixed-time AI, cow

## Abstract

**Simple Summary:**

The magnitude and duration of the pre-ovulatory release of LH is important in controlling the timing of ovulation in estrus synchronization and fixed-time artificial insemination (FTAI) protocols in cows. The neuropeptide kisspeptin (KISS1) stimulates GnRH neurons in the brain which in turn induce LH release from the pituitary gland. The present study used ovariectomized Nelore cows as a model to examine whether priming with the steroid estradiol benzoate (EB) for 12 h increased both the peak and duration of LH release after treatment with KISS1. It was found that cows pre-treated with EB for 12 h showed a greater LH peak and longer duration of LH release in response to KISS1 compared with cows that received EB at the same time as KISS1, or KISS1 alone. The incorporation of EB priming and KISS1 in estrus synchronization-FTAI protocols could improve the control of LH release and ovulation, and potentially increase fertilization and conception rates in cows. This would have important practical application in assisted breeding in beef and dairy cows.

**Abstract:**

The present study examined whether priming with estradiol benzoate (EB) for 12 h increased both the peak and duration of LH release in response to kisspeptin (KISS1, KP) in cows. In a Latin square design, ovariectomized Nelore cows (*n* = 8) received: Control, i.m. 4 mL of 0.9% saline; KP, i.m. 4 mg murine KISS1-10; EBKP, i.m. 4 mg KISS1-10 + i.m. 2 mg EB simultaneously; EB12KP, i.m. 4 mg KISS1-10 + i.m. 2 mg EB 12 h before KISS1-10. Concentrations of LH were determined in blood samples obtained at time 0 (treatment), 20, 40, 60, 90, 120, 150, 180, 210 and 270 min. Concentrations of LH were analyzed by Proc GLIMMIX for repeated measures. In case of significance, the adjusted Tukey test was used to test for differences among treatments. GraphPad 8.0 PRISM^®^ was used to determine the area under the LH-response curve (AUC) after injection of KISS1-10. Plasma LH remained relatively constant throughout sampling after treatment with saline. The peak in LH after injection of KISS1-10 occurred at 20 min in Groups KP and EBKP and at 40 min in Group EB12KP. The peak LH response (∆LH, ng/mL) was greater (*p* < 0.01) in Group EB12KP (5.6 ± 0.9) than in Groups KP (2.4 ± 0.9) and EBKP (3.5 ± 0.9), which did not differ. AUC (LH ng/mL*min) was greater (*p* = 0.02) in Group EB12KP (439 ± 73) than in Groups KP (176 ± 73) and EBKP (241 ± 73), with the latter two groups not differing. The findings indicated that 12 h priming with EB increased both the peak and duration of the LH response to treatment with KISS1. The incorporation of EB priming and KISS1 could improve the efficiency of estrus synchronization with fixed-time AI in cows. This would have an important practical application in assisted breeding in beef and dairy cattle.

## 1. Introduction

Synchronization of the stage of the estrous cycle and ovulation, combined with fixed-time artificial insemination (FTAI), has proven to be a powerful technology platform to improve both reproductive performance and the rate of genetic gain in beef and dairy cattle [1]. The adoption of assisted reproductive technology has increased rapidly as the beef and dairy industries have sought to develop efficient and sustainable production systems to meet the growing demand for animal protein [2]. The large-scale adoption of assisted breeding requires that the technology has a high level of success and is optimally cost-effective [3]. Even modest gains in the efficiency of assisted breeding can have major impact when applied across the beef and dairy industries.

The synchronization of ovulation in preparation for FTAI remains an area where further improvements can be made. Critical to the control of ovulation are the magnitude and timing of the pre-ovulatory surge release of LH [4]. Hormones that are most commonly used to influence the timing of the LH surge include GnRH [5] and estradiol (E2) [6]. Other hormones that directly influence the maturation and ovulation of ovarian follicles are human chorionic gonadotropin (hCG) [7] and equine chorionic gonadotropin (eCG) [8]. All of these hormones are used singularly and in different combinations, as summarized in several reviews [9,10,11,12]. More recently, kisspeptin (KISS1) has been added to hormones that can be used to stimulate the release of LH in cattle [13].

An outstanding issue in synchronization–FTAI protocols is whether greater precision can be achieved in both the timing and magnitude of LH release. An improvement in the control of LH release should reduce the variability in the interval from treatment to ovulation [14]. This would increase the likelihood of fertilization at FTAI. A combination of KISS1 and E2 provides two potential mechanisms for the control of LH release from gonadotrope cells in the anterior pituitary gland. In this approach, E2 would have a priming effect on gonadotrope cells which should enhance their sensitivity and response to GnRH. KISS1 could stimulate the release of LH through two pathways. One pathway is the established action of KISS1 at GnRH neurons in the hypothalamus [15]. A second potential pathway could be a direct action of KISS1 on gonadotrope cells [16]. Gonadotropes have KISS1 receptor GPR54 [17] and KISS1 induced LH release from cultured bovine anterior pituitary cells [18]. However, KISS1 receptor GRP54 also occurs on pituitary lactotrope and somatotrope cells and it remains unclear whether KISS1 action at the gonadotrope cells is an important component of LH release [19]. Notwithstanding, the above KISS1 + E2 rationale was used in the present study to examine LH release in OVX cows treated with KISS1, either alone or in combination with E2. OVX cows were used in the present study to exclude the endogenous source of ovarian steroids. Ovarian E2 and P4 have a feedback action in the brain which regulates the release of GnRH and LH in cows [20]. It was important to use a model in the present study which excluded a potential effect of endogenous E2 and P4 on the response to KISS1. The hypothesis tested was that pre-exposure to E2 would have a priming effect and increase the LH response to KISS1 in cows. This information could provide new insight on the mechanisms that control LH release in cattle with potential application in estrus synchronization–FTAI protocols in beef and dairy cows.

## 2. Materials and Methods

### 2.1. Animals and Experimental Procedures

The study had approval from the Ethics Committee of the University of Sao Paulo, Sao Paulo, Brazil (protocol #2537/2012). Nelore (*Bos indicus*) cows (*n* = 8; 3-y-old; 432 ± 27 kg) were used. They were maintained on pasture that was predominant in palisade grasses (*Brachiaria brizanta*) and had free access to mineralized salt and water. All cows underwent bilateral ovariectomy (OVX) using an established L-block flank laparotomy technique [21]. Briefly, the flank was incised using aseptic conditions and the subcutaneous and muscular layers were separated to gain access to the peritoneal cavity. After removal of both ovaries, the peritoneum, muscle layer, and skin, were sutured separately. Cows were monitored for 24 h after surgery and the wounds checked at 3 and 7 days.

**Figure 1:**
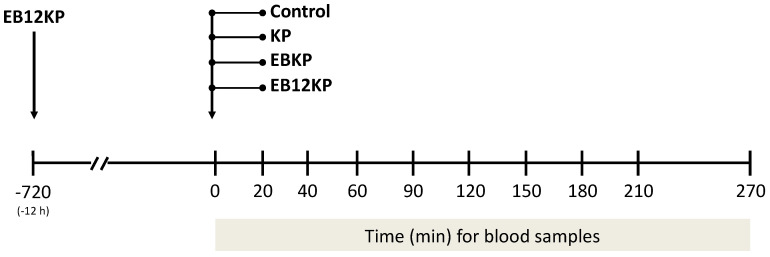
Study design. Ovariectomized Nelore cows (*n* = 8) were used in a Latin square design and received the following treatments: Control, i.m. 4 mL 0.9% saline; KP, i.m. 4 mg murine KISS1-10 in 4 mL 0.9% saline; EBKP, i.m. 4 mg KISS1-10 + i.m. 2 mg estradiol benzoate (EB) simultaneously; EB12KP, i.m. 2 mg EB 12 h before i.m. 4 mg KISS1-10. Blood samples were obtained at 0, 20, 40, 60, 90, 120, 150, 180, 210, and 270 min relative to the time of treatment.

One month after ovariectomy, the eight cows were used in a Latin square experimental design. The treatments were as follows: (1) Control, cows received i.m. 4 mL 0.9% saline; (2) KP, cows received i.m. 4 mg murine KISS1-10 in 4 mL 0.9% saline; (3) KP + estradiol benzoate (EBKP), cows received i.m. 4 mg KISS1-10 + i.m. 2 mg EB (Sincrodiol^®^, Ourofino, Brazil) simultaneously; (4) EB12KP, cows received i.m. 2 mg EB 12 h before i.m. 4 mg KISS1-10 (Figure 1). At days 0, 10, 20 and 30, two cows were randomly assigned to each treatment so that at the end of the experiment all cows had received all treatments (*n* = 8/treatment), and no cow received a single treatment twice. The dose of KISS1-10 was intended to give approximately 10 µg KISS1-10/kg BW based on a previous report [13]. The EB dose was also based on previous studies [6]. Murine KISS1-10 decapeptide was obtained by custom synthesis (American Peptides Company, Inc., Sunnyvale, CA, USA). The KISS1-10 peptide was 96.5% pure based on HPLC analysis and was of molecular mass unit 1318.4 based on mass spectral analysis.

Blood samples (8 mL) were obtained by jugular venipuncture to determine circulating concentrations of LH. Samples were taken at 0, 20, 40, 60, 90, 120, 150, 180, 210, and 270 min relative to the time of treatment (Figure 1). A sampling interval of 20–30 min was chosen based on the established nature of pituitary LH release in cattle which is characterized by random pulses and a half-life in circulation of 20–30 min. Blood was collected into evacuated tubes containing sodium heparin (Vacutainer^®^; Becton-Dickinson & Company, Franklin Lakes, NJ, USA) and placed on ice until centrifugation (1500× *g* for 20 min, 4 °C). Plasma was stored at −20 °C until processed for LH and progesterone (P4) radioimmunoassay (RIA).

### 2.2. Hormone Quantification

Concentrations of P4 in circulation were measured to confirm that the OVX had removed ovarian tissue and that there was no endogenous source of P4 in the cows. Endogenous P4 would influence the responses to treatment with E2 and KISS1. The P4 radioimmunoassay used a commercial kit (Coat-A-Count, Diagnostics Products Co., Los Angeles, CA, USA) with sensitivity of 0.05 ng/mL. Samples were analyzed for P4 in the one assay and the intra-assay coefficient of variation was 4.3%. LH was quantified by a liquid phase double antibody radioimmunoassay as described previously [22]. Highly purified LH (AFP8614B; National Hormone and Pituitary Program, Rockville, MD, USA) was used to prepare both the radioiodinated LH and reference LH standards. The sensitivity of the assay was 0.05 ng/mL. The intra- and inter-assay coefficients of variation were 10.3% and 13.1%, respectively.

### 2.3. Statistical Analyses

The program GraphPad 8.0 PRISM^®^ (https://www.graphpad.com/guides/prism/8/user-guide/index.html, accessed on 25 April 2021) was used to identify pulses of LH and to determine the following LH secretory characteristics: basal concentration; area under the LH-response curve (AUC, LH released from the beginning until the end of the LH peak after injection of KISS1-10); and ∆LH (peak LH concentration after injection of KISS1-10 minus the concentration at 0 min). Statistical analysis was performed with the Statistical Analysis System software for IOS (SAS^®^ University Edition; https://www.sas.com/en_au/software/university-edition/download-software.html, accessed on 24 November 2020). Variables were assessed with the UNIVARIATE procedure for normality of residuals by the Shapiro-Wilk test, and for homogeneity of variances with the Bartlett test. AUC and ∆LH did not show normality of residuals and the data were transformed to Log-10 before analysis. Concentrations of LH were analyzed by Proc GLIMMIX for repeated measures. Concentrations of P4 at time 0 were analyzed by Proc GLIMMIX. In case of significance, the adjusted Tukey test was used to test for differences among treatments. A 5% significance level was used, and the data are presented as estimated means ± SEM.

## 3. Results

### 3.1. Progesterone

Concentrations of P4 in circulation were low (0.43 ± 0.08 ng/mL, treatments combined) as would be predicted for OVX cows that lack ovaries to secrete P4. There was no difference (*p* = 0.87) in P4 among treatment groups.

### 3.2. LH

**Figure 2:**
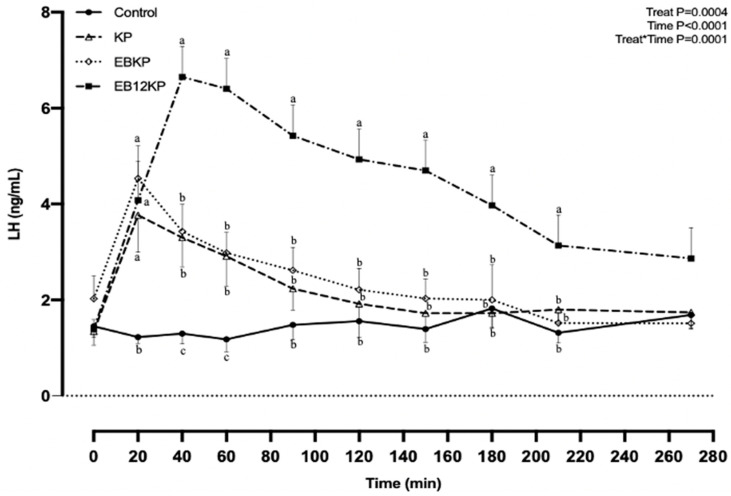
Plasma LH concentrations (means and sem) in ovariectomized Nelore cows (*n* = 8) that received the following treatments: Control, i.m. 4 mL 0.9% saline (Control) at time 0 min; KISS1-10 (KP), i.m. 4 mg murine KISS1-10 in 4 mL 0.9% saline at time 0 min; EBKP, i.m. 4 mg KISS1-10 + i.m. 2 mg estradiol benzoate (EB) simultaneously at time 0 min; EB12KP, i.m. 2 mg EB 12 h before i.m. 4 mg KISS1-10 at time 0 min. Treatment, time, and treatment*time effects were highly statistically significant. ^a,b,c^ LH concentrations within time points without a common letter differ (*p* < 0.05).

Concentrations of LH in circulation before treatment with KISS1-10 (time 0 min) did not differ (*p* = 0.89) among the treatment groups (Control, 1.45 ± 0.65; KP, 1.34 ± 0.65; EBKP, 2.02 ± 0.65; EB12KP, 1.39 ± 0.65 ng/mL). LH remained relatively constant from 0 to 270 min when cows received 0.9% saline and served as controls (Figure 2).

Treatment with KISS1-10 consistently induced a release of LH (*p* = 0.0135). The highest concentration of LH occurred at 20 min in Groups KP and EBKP (Figure 2). Group EB12KP had the highest concentration of LH at 40 min. The amplitude of the LH response (∆LH) was similar for Groups KP and EBKP, which both had a lesser (*p* < 0.01) LH amplitude response than Group EB12KP (Table 1).

In Groups KP and EBKP, LH remained above pre-treatment levels for 90 min, whereas Group EB12KP continued to have levels of LH above pre-treatment at 270 min (Figure 2). The greater amplitude of the LH response for Group EB12KP, combined with continued elevated LH, meant that the area under the LH-response curve (AUC) for this group was greater (*p* = 0.02) than for Group KP and Group EBKP, with the latter two groups not differing (Figure 2, Table 1).

## 4. Discussion

The hypothesis tested in the present study was that pre-exposure to E2 would have a priming effect and increase the LH response to KISS1 in OVX cows. Cows exposed to EB for 12 h before treatment with KISS1 showed a greater amplitude in the LH response and longer duration of elevated LH compared with cows treated with EB at the time of KISS1, or cows treated with KISS1 alone. Based on this finding, it was concluded that pre-treatment with EB primed the gonadotrope cells in the anterior pituitary to respond to GnRH. The present study did not include a group treated with EB alone. Whilst this might be considered an omission, the LH response in cows to treatment with EB has been well characterized in previous studies [6,23]. The inclusion of an EB group would not have explained the differences in LH response between cows that received EB 12 h before KISS1 or in conjunction with KISS1. The 12 h interval between pre-treatment with EB and treatment with KISS1 was based on the report that maximal pituitary GnRH receptors in cows were observed 12 h after treatment with E2-17β [24]. OVX cows also showed a greater LH response to GnRH when treatment was started 8 h after an injection of E2-17β compared with 2 h after an injection of E2-17β [25]. In the present study, KISS1 would have stimulated GnRH neurons in the hypothalamus to release GnRH which then acted at E2-primed gonadotrope cells in the pituitary. A second potential pathway for KISS1-induced LH secretion is a direct action of KISS1 at gonadotrope cells. As noted above, gonadotrope cells have GPR54 [17] and KISS1 stimulated LH release by bovine pituitary cells in culture [18]. However, a direct action of KISS1 at gonadotropes in vivo remains controversial and requires further study.

The LH response at 20 min was similar for cows that received EB either 12 h before KISS1 or in conjunction with KISS1. This observation could be interpreted to suggest that the immediately releasable pool of LH [26] was the same for these two groups of cows and was independent of E2 priming and gonadotrope cell GnRH receptor numbers. The E2 priming response was manifested as a greater amplitude and longer duration of the LH response to KISS1. These two features of LH secretion in E2-primed animals would be expected to increase both the proportion of cows that ovulate and the synchrony of ovulation in cows enrolled in a synchronization–FTAI protocol [20].

GnRH neurons have the KISS1 receptor GPR54 and also neuropeptide Y1 receptor (Y1R) [27] and melanocortin receptor (MCR) [28]. Receptor Y1R is linked to NPY/AgRP (NPY) neurons and receptor MCR is linked to pro-opiomelanocortin (POMC) neurons. NPY and POMC neurons are modulated by nutritional status and metabolic condition and both neurons can either stimulate or suppress GnRH neurons [29,30]. NPY and POMC neurons have E2 receptor-α (ERα) and E2-17β can inhibit these neurons [31,32]. In the present study, EB administered 12 h before KISS1 may have suppressed NPY and POMC neurons and reduced their negative action at GnRH neurons. This would have allowed GnRH neurons to undergo a greater response to KISS1. This potential scenario illustrates the complexity of mechanisms and pathways that can influence GnRH-LH secretion. A deeper understanding of this biology could lead to further improvements in synchronization–FTAI protocols.

In previous studies, KISS1 was administered by intravenous (i.v.) or intracerebroventricular (i.c.v.) injection in rodents [33,34,35,36,37], sheep [15,38], primates [36,39], cattle [37] and pigs [40]. A study in calves utilized i.m. KISS1 [13]. The latter study, together with the present findings, showed that i.m. injection of KISS1 induces a release of LH that is comparable to other routes of KISS1 administration. This makes KISS1 practical for potential widespread adoption in synchronization–FTAI protocols. In countries where E2 is not registered for use in cattle, KISS1 agonists with a long half-life in circulation could be a practical option for the induction of LH release in cows [41].

## 5. Conclusions

Priming with E2 for 12 h increased both the amplitude and the duration of the LH response after i.m. injection of KISS1 in ovariectomized Nelore cows. The incorporation of E2 priming and KISS1 in estrus synchronization-FTAI protocols could improve the control of LH release and ovulation in cows. This has potential to increase fertilization and conception rates in cows enrolled in estrus synchronization-FTAI programs. There are important implications for assisted breeding and genetic improvement in beef and dairy cattle, and other livestock. Further research is needed to optimize the dose of KISS1 that induces an optimal release of LH for ovulation in cattle.

## Figures and Tables

**Table 1:** Parameters of LH release in ovariectomized Nelore cows treated i.m. with 4 mg murine KISS1-10 (KP); KISS1-10 + i.m. 2 mg estradiol benzoate simultaneously (EBKP); and KISS1-10 + i.m. 2 mg EB 12 h before KISS1-10 (EB12KP). AUC (LH ng/mL*min), area under the LH-response curve; ∆LH (ng/mL), highest LH concentration minus the LH concentration at time 0 min (treatment). Results are means ± sem.

	KP	EBKP	EB12KP	*p*-Value
AUC (ng/mL*min)	176 ± 73 ^a^	241 ± 73 ^a^	439 ± 73 ^b^	0.02
△LH (ng/mL)	2.4 ± 0.9 ^a^	3.5 ± 0.9 ^a^	5.6 ± 0.9 ^b^	<0.01

^a,b^ means within rows without a common superscript differ.

## Data Availability

Not applicable.
